# Designing of a penta-peptide against drug resistant E. coli

**DOI:** 10.6026/97320630013192

**Published:** 2017-06-30

**Authors:** Sachin Nagra, Deepak Kumar, Rajasri Bhattacharyya, Dibyajyoti Banerjee, Tapan Mukherjee

**Affiliations:** 1 Department of Biotechnology, Maharishi Markandeshwar University, Mullana, Ambala, haryana 133207; 2 Department of Experimental Medicine and Biotechnology, Postgraduate Institute of Medical Education and Research, Chandigarh 160012; 3 past: Department of Biotechnology, Maharishi Markandeshwar University, Mullana, Ambala, haryana 133207; present: Department of Experimental Medicine and Biotechnology, Postgraduate Institute of Medical Education and Research, Chandigarh 160012

**Keywords:** antibiotic resistant, penicillin binding protein, antimicrobial peptide, peptide designing

## Abstract

Drug resistant pathogens are vibrant global problem. Penicillin binding protein 5 (PBP5) plays important role in bacterial cell wall biosynthesis. Mutation in PBP5 is a well-known mechanism for development of drug resistant strain of bacteria. In this context we have designed a peptide that fits better at the ligand-binding site of mutant PBP5 compared to wild type PBP5. It is expected that the designed peptide will halt the growth of drug resistant pathogen harboring mutant variety of PBP5. We have recommended experimental validation of the above concept.

## Background

Antibiotic resistance is a burning global problem [1]. In evolution of antibiotic resistance point mutations in the bacterial genome or genetic mechanisms play crucial role [2,3]. The problem of antibiotic resistance is so critical that at the present moment the world is threatened by the emergence of multi drug resistant strains of bacteria [4]. Penicillin binding protein (PBP) plays important role in bacterial cell wall biosynthesis through its trans-glycosylation and trans-peptidation functions [5]. Substitution in PBP is a recognized mechanism in genesis of antibiotic resistance strains of pathogenic bacterium [6]. Antimicrobial peptides like gramicidin A are successful antibiotics [7]. This is an upcoming field of research that has gained considerable research interest [8]. In this work a novel antimicrobial peptide is designed that binds with a mutated PBP better than wild type PBP. This approach may pave the path for novel antimicrobial peptide development against drug resistant pathogens.

## Methodology

### Three-dimensional structures of PBP5, antibiotics and peptide

**Figure 1:**
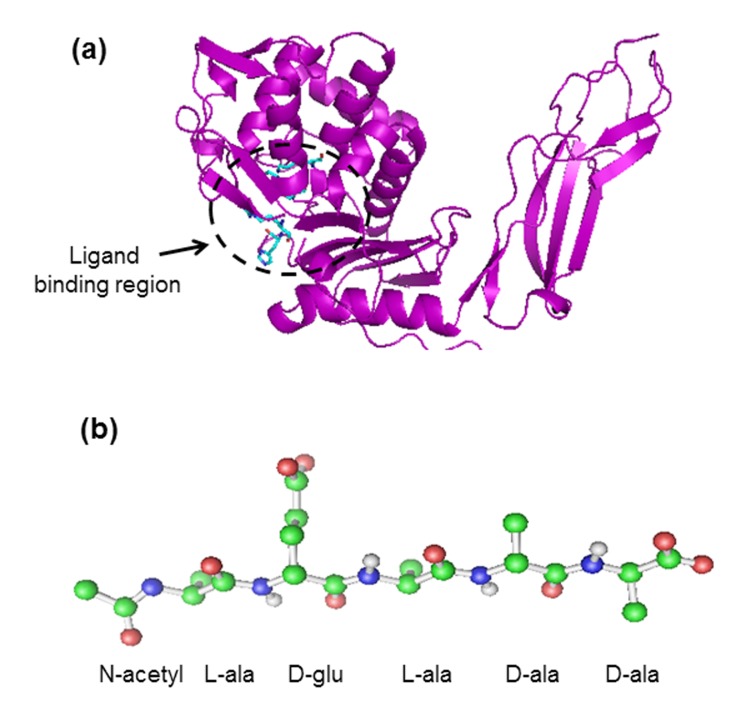
(a) The three dimensional structure of PBP5 is shown in cartoon representation. The ligand-binding region is encircled. (b) The peptide sequence is represented in ball-and-stick mode. The positions of the amino acids are labelled.

Three-dimensional structures of wild type and mutant (Gly105asp) PBP5 of E. coli (PDB code 1NZO and 1NJ4, respectively) were obtained from RCSB Protein Data Bank [9]. In Figure 1(a) the cartoon representation along with ligand binding site of PBP5 is shown. Three-dimensional structures of the antibiotics - penicllin, ampicillin, tetracycline and chloramphenicol were downloaded from NCBI Pubchem [10]. Discovery studio 3.1 was used for building the peptide with sequence Lala-Dglu-Lala-Dala-Dala. To model the peptide build and edit protein tool was used. D-amino acids were modeled by stereochemistry tool under Chemistry main menu. The N-terminus of the peptide was also capped by acetylate group. Then the energy of the peptide was minimized. In Figure 1(b) the ball-and-stick representation of the peptide is given. Active site residues of PBP5 i.e Ser44, Lys47, Ser86, Ser87, Gly105, Gln 109, Ser110 and Arg198 were identified by literature study [11, 12, 13].

### Docking of antibiotics and peptide at the ligand binding site of PBP5

Penicillin, ampicillin, tetracycline and chloramphenicol were docked using Autodock4.2.6 docking tool [14] at the binding pocket of PBP5. At first all the side-chain functional atoms were considered as grid center individually for docking of penicillin with PBP5. It was observed that grid center on OG atom of Ser110 and grid size 60X60X60 xyz points with grid spacing 0.375 Å give the best result. Because here maximum number of active site residues of PBP5 were in close contact with penicillin. So other antibiotics were docked by considering OG of Ser110 as grid center. In the same way the peptide was also docked at the penicillinbinding site of wild type and mutant protein. Mutations of Ser44 of wild type protein by Gly and Cys were done in pymol [15]. Energy minimizations of these mutated structures were performed by energy minimization tool of swisspdbviewer [16]. Conformation having highest negative binding energy obtained from autodock was considered for analysis purpose. Pymol structure visualization software [15] was used to visualize the docked complex and to calculate the non-bonded distances between antibiotic and different active site residues.

## Results

**Figure 2:**
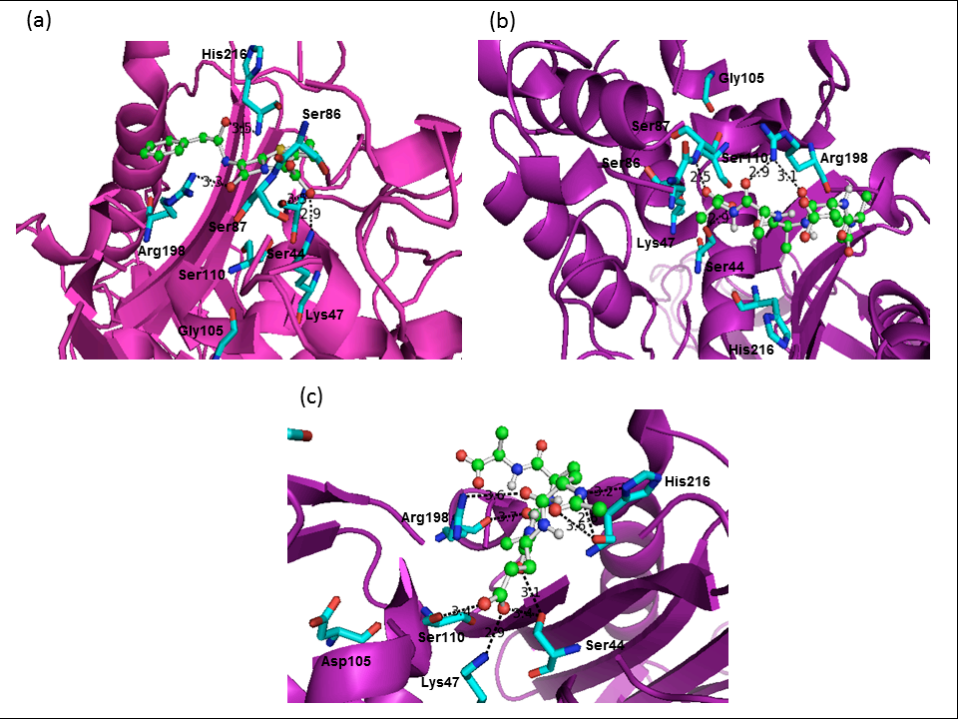
Interaction of penicillin with PBP5 (a), designed peptide with PBP5 (b) and designed peptide with Gly105asp mutant PBP5 (c) are shown. The penicillin and the peptide are represented by ball-and-stick mode (carbon atoms in green color) while the interacting residues of wild/mutant PBP5 are shown in stick mode along with residue identifier. The hydrogen bonds are represented by dotted lines with distance mentioned.

The active site residues of PBP5 those are in close vicinity of penicillin are mentioned in figure 1a. Among them, Ser44, Lys47, Arg198 and His216 are observed to be within the hydrogen bond distances (less than or equal 3.5 Å) [17]. At the ligand-binding site, the b-lactum ring is positioned in such a way that there is chance of hydrogen bond formation between carbonyl oxygen (oxyanion) - Arg198 and carboxylate - ser44. Moreover, the acyl group is also observed in close contact with ligand binding His216. The binding energy of penicillin with PBP5 is -7.06 kcal/mol. Ampicillin, tetracyclin and chloramphenicol has also resembled the same interaction pattern with PBP5. The binding energies of ampicillin and tetracyclin are observed to be more negative (-7.84 and -7.78 kcal/mol, respectively). However, in peptide-PBP5 complex, the binding orientation of the peptide at the ligand-binding site is different (Figure 2b). Only Ser44, Ser110 and Arg198 are observed to be within the hydrogen bond distance and the orientation of the peptide is observed to be away from the oxyanion hole comprising Ser44 and His216 [18]. Here the binding energy is also less (-5.79 kcal/mol). In Gly105asp mutant PBP5, the active site residues those are in contact with penicillin (Figure 2a) are observed to be in contact with the peptide (Figure 2c). The ND1 of His216 is also observed in contact with peptide. The binding energy is found to be more negative (-5.99 kcal/mol) as compared to peptide-wild type PBP5 complex. In Ser44-cys and Ser44-gly mutated PBP5 proteins, the peptide is not fitted at the ligand binding site and in both complexes the binding energies are observed in the positive range (around 3.89 kcal/mol) (data not shown).

## Discussion

The designed peptide has sequence similarities with peptide portion of bacterial cell wall peptidoglycan. Therefore, it is expected that it will fit at the ligand-binding site of PBP5 because PBP5 catalyzes the transglycosylation as well as cleavage of DAla- DAla through transpeptidase activity and cross-linking of glycan strands. It is observed that the designed peptide is interacting with all the ligand binding residues of PBP5 analogous to penicillin (Figure 2). This hints that the designed peptide may have penicillin like antimicrobial activity. Interestingly, in Gly105asp mutant PBP5, His216 that is an important residue of PBP5 for ligand binding, the ring nitrogen is also in contact within hydrogen bond distance with the designed peptide. The above phenomenon is not observed in peptide-wild type PBP5 complex. Furthermore, with the above-referred mutant type of protein the designed peptide is interacting with more favorable binding energy compared to wild type. All these have implications that the designed peptide will interact better with His216 of the mutant PBP5 compared to the wild type. This means that the designed peptide has the potential to block the cell wall biosynthesis of the bacteria containing the mutant type of the protein. In other words the peptide has the potential to act as an antibiotic for the particular drug resistant pathogen expressing the mutant type of PBP5. This peptide is expected to hinder the growth of normal flora containing wild type of the protein comparatively less than the mutant type and so antibiotic associated adverse drug reactions (like pseudo-membranous collitis) are expected to be less. We feel that experimental verification of the predicted results is necessary in days to come.

## Conclusion

In this study a peptide is designed which has potential antimicrobial action on drug resistant bacterial strain because it binds better with mutant PBP5 compared to wild type.
